# Functional characterization of LSM12 as a driver in uveal melanoma oncogenesis

**DOI:** 10.1016/j.aopr.2025.09.001

**Published:** 2025-09-10

**Authors:** Junjie Tang, Fengyu Sun, Yi Ren, Liling Chen, Yang Gao, Jinmiao Li, Yaoming Liu, Chao Cheng, Ping Zhang, Shuxia Chen, Siming Ai, Yuxiang Mao, Shicai Su, Rong Lu

**Affiliations:** a State Key Laboratory of Ophthalmology, Zhongshan Ophthalmic Center, Sun Yat-Sen University, Guangdong Provincial Key Laboratory of Ophthalmology and Visual Science, Guangzhou, China; b Department of Ophthalmology and Otorhinolaryngology, The Baiyun Hospital of Guangzhou First People's Hospital (the Second People's Hospital of Baiyun District), Guangzhou, China

**Keywords:** Uveal melanoma, LSM12, Cancer biomarker, PI3K/AKT/mTOR

## Abstract

**Objective:**

This study aimed to investigate the role of LSM12 in uveal melanoma (UM) oncogenesis and progression, examining its potential as both a prognostic biomarker and therapeutic target.

**Methods:**

LSM12 expression was analyzed in relation to RNA modification genes and tumor stemness in UM. UM cell lines were subjected to LSM12 knockdown using siRNA, followed by cell viability and migration assays. The therapeutic potential of targeting LSM12 was evaluated using a subcutaneous xenograft model. Additionally, the relationship between LSM12 and the PI3K/Akt/mTOR pathway was explored.

**Results:**

LSM12 expression levels were significantly elevated in UM patients, correlating strongly with poor prognosis. Positive correlations were observed between LSM12 expression and multiple genes associated with RNA methylation modifications and cancer stem cell characteristics. Knockdown of LSM12 effectively disrupted UM cell viability and migration in vitro and inhibited OCM1 xenograft growth in vivo. Additionally, LSM12 knockdown resulted in notable inhibition of the PI3K/Akt/mTOR pathway both in vitro and in vivo.

**Conclusions:**

Elevated LSM12 expression correlates with poor prognosis in UM and critically promotes oncogenic processes, including tumor cell viability, migration, and tumorigenesis.

## Highlights

• LSM12 expression is significantly elevated in UM and correlates with poor patient prognosis.
• Genetic knockdown of LSM12 inhibits UM cell viability, migration, and in vivo tumor growth.
• LSM12 promotes UM progression via activation of the PI3K/Akt/mTOR signaling pathway.

## 1 Introduction

Uveal melanoma (UM) is the most prevalent primary intraocular malignancy in adults. Originating in the uveal tract of the eye, UM is a rare but aggressive and lethal neoplasm characterized by a unique biology and clinical phenotype distinct from the cutaneous melanoma.^1^ While radiotherapy, enucleation, and other treatments achieve local control in over 90% of cases, more than 40% of patients eventually develop distant metastases, primarily in the liver.^2^, ^3^, ^4^ Recent research has significantly advanced the understanding of the genetics, genomics, and molecular alterations in UM. These investigations have identified critical genetic aberrations, which have enhanced tumor classification and provided more accurate prognostication of metastatic disease.^5^ Among these findings, epigenetic changes have been shown to contribute to the rapid progression of UM following classic genetic alterations.^6^ Notably, the druggable potential of epigenetic modifications provides a distinct avenue for innovating new therapeutic options for UM. Therefore, expanding the understanding of epigenetic regulation markers in UM is crucial for advancing epigenetic-directed therapies.

Smith-like (LSM) proteins are part of a large family involved in various RNA processing and gene expression regulation processes. Each LSM protein monomer is multifunctional, contributing to diverse cellular functions.^7^^,^^8^ Recent studies have demonstrated that LSM family members play critical roles in oncogenesis, particularly through their involvement in RNA processing events across several types of malignancies.^9^, ^10^, ^11^ LSM12, a member of the Sm family, plays a key role in RNA splicing, and aberrant RNA splicing is recognized as a contributor to carcinogenesis.^12^ Notably, studies on UM have found that splicing factor mutations lead to abnormalities in the alternative splicing of long non-coding RNAs (lncRNAs).^13^ Additionally, recent research has shown that LSM12 can regulate the alternative splicing of USO1 exon 15, which is closely related to the malignant phenotypes of oral squamous cell carcinoma cells.^14^ However, the biological function of LSM12 in UM tumorigenesis remains unclear.

Thus, this study integrated database analysis with cellular and animal experiments to investigate the role of LSM12 in UM tumorigenesis. The findings from this research may elucidate the precise molecular mechanisms for targeted treatments in UM.

## 2 Materials and methods

### 2.1 Clinical samples

This study was approved by the Ethics Committee of Zhongshan Ophthalmic Center (2023KYPJ308) and adhered to the Declaration of Helsinki. A single paraffin-embedded tissue section from enucleated eyeball of a 60-year-old male with pathologically confirmed unilateral UM was retrieved from the Department of Pathology for immunohistochemical (IHC) validation of LSM12 expression. The sample was stored at room temperature in the institutional pathology archive under standard conditions post-diagnosis, ensuring integrity for subsequent analysis.

### 2.2 Cell culture

The human retinal pigment epithelial cell line ARPE-19 (ATCC CRL-2302, USA), the OCM1 cell line (amelanotic subtype, lacking melanin pigmentation, provided by the Department of Ocular Oncology at Zhongshan Ophthalmic Center), and the human uveal melanoma cell line MEL270 (CellCook CC1806, China) were each cultured in Dulbecco's Modified Eagle Medium/Nutrient Mixture F-12 (DMEM/F-12, Gibco, Thermo Fisher Scientific, USA) or RPMI 1640 medium (Corning, USA), both supplemented with 10% fetal bovine serum (FBS, Gibco, Thermo Fisher Scientific, USA) and 1% penicillin-streptomycin (Invitrogen, USA). All cell lines were authenticated by short tandem repeat (STR) profiling and routinely tested for mycoplasma. Cultures were incubated at 37 ​°C in a humidified atmosphere containing 5% CO_2_.

### 2.3 Data mining of public datasets

Survival analysis data and pathway activity analysis data were obtained from The Cancer Genome Atlas (TCGA) using the Gene Set Cancer Analysis (GSCA) platform.^15^ Analyses of RNA modification genes (m1A, m5C, m6A) and the relationship between tumor stemness and gene expression were performed using standardized pan-cancer datasets from UCSC (https://xenabrowser.net).^16^ Tumor stemness, specifically the DNA methylation-based tumor stemness score, was computed for each tumor by integrating sample-specific stemness indices with gene expression data, which were then logarithmically transformed (log_2_(x+0.001)) as previously described.^17^ LSM12 expression levels across different stages of uveal melanoma were retrieved from UALCAN (https://ualcan.path.uab.edu).^18^^,^^19^ The TIMER correlation module was used to evaluate potential relationships between LSM12 expression and the PI3K-AKT-mTOR pathway (https://cistrome.shinyapps.io/timer).^20^

### 2.4 Histology and immunohistochemical analyses

Hematoxylin and eosin (H&E) staining and immunohistochemistry (IHC) were performed on 4-μm-thick paraffin sections of human or mouse tissues using standard protocols.^21^ Human UM samples were subjected to H&E staining for histopathological evaluation. The expression of LSM12 (1:500, Proteintech, 67029-1-Ig), Ki67 (1:2000, Proteintech, 27309-1-AP), pAKT (1:50, Zenbio, 381555), and pmTOR (1:50, Zenbio, R25033) was assessed by IHC. Staining was evaluated semi-quantitatively based on the proportion of positively stained tumor cells.

### 2.5 Western blotting

Cells were lysed in RIPA buffer supplemented with protease and phosphatase inhibitors. Protein lysates were analyzed by Western blotting to detect LSM12 (1:2000, Proteintech, 67029-1-Ig), GAPDH (1:10000, Proteintech, 10494-1-AP), MMP9 (1:1000, Zenbio, 380831), N-cadherin (1:1000, Zenbio, 380671), E-cadherin (1:1000, Zenbio, 340341), pPI3K (1:1000, Zenbio, 341468), pAKT (1:1000, Zenbio, 381555), and pmTOR (1:1000, Zenbio, R25033).

### 2.6 RNA extraction and quantitative real-time PCR (qPCR)

Total RNA was extracted from cells using the RNA Purification Kit (B0004D, EZBioscience) according to the manufacturer's instructions. cDNA synthesis was performed with 1000 ​ng of total RNA using the EZBioscience® 4 ​× ​Reverse Transcription Master Mix (A001GQ). PCR amplification was conducted in a 10 ​μl reaction. GAPDH served as the reference gene, and relative gene expression levels were calculated using the 2-ΔΔCT method. Experiments were performed in triplicate. Primer sequences were: GAPDH Forward: *GGAGCGAGATCCCTCCAAAAT*, GAPDH Reverse: *GGCTGTTGTCATACTTCTCATGG*; LSM12 Forward: *CTGTTCTTCCCACCTCA*, Reverse: *GCACACTGGCTCTACAAA*.

### 2.7 siRNA transfection

RNA interference was used to knockdown LSM12 expression. Three pairs of small interfering RNAs (siRNAs) were designed for this purpose, with one pair targeting LSM12 (siLSM12; sense: 5′-*GUAUGUUUCAGAAGUGGAATT*-3′, antisense: 5′-*UUCCACUUCUGAAACAUACTT*-3′) and a non-targeting negative control siRNA (siNC; sense: 5′-*UUCUCCGAACGUGUCACGUTT*-3′, antisense: 5′-*ACGUGACACGUUCGGAGAATT*-3′) being synthesized by GenePharma Co., Ltd. The sequences for the other two siRNA pairs are listed in supplementary material. Cells were transfected with 50 ​nM siRNA using HiPerFect Transfection Reagent (301704, Qiagen) following the manufacturer's instructions.

### 2.8 Cell viability assay

Cell viability of MEL270 and OCM1 cells was determined using the Cell Counting Kit-8 (CCK-8, A311-01, Vazyme Biotech Co., Ltd.). Cells were seeded into 96-well plates at a density of 3000 ​cells per well and then treated with siLSM12. Following the treatment period, 10 ​μl of CCK-8 solution was added to each well. After a 4-h incubation at 37 ​°C, the absorbance was quantified at 450 ​nm using a microplate reader. All measurements were conducted in triplicate.

### 2.9 Cell colony formation assay

Following transfection, MEL270 and OCM1 cells were plated in 6-well plates at a density of 1 ​× ​10^5^ ​cells per well and cultured for 24 ​h, with medium replaced every 3 days. After 7 days, cells were stained with 0.1% crystal violet for 1 ​h. Clusters of more than 50 ​cells were counted as colonies. The entire experiment was conducted in triplicate.

### 2.10 Flow cytometry

Cells were seeded into 6-well plates at a density of 100,000 ​cells per well and treated with siRNA. Cells were harvested, washed with cold PBS. After resuspension and rehydration with PBS, cells were stained with propidium iodide and annexin V, incubated for 30 ​min in the dark at room temperature, and analyzed using a LSRFortessa flow cytometer (BD Bioscience, USA).

### 2.11 Scratch test

Cell migration was assessed using a scratch wound healing assay. A confluent cell monolayer was scratched with a sterile 10-μl pipette tip. After washing the cells with PBS to remove cell debris, the cells were cultured in fresh medium for 18 ​h. The wound gap was monitored, and the average distance of wound closure was measured at 0 and 18 ​h.

### 2.12 Transwell assay

Transwell inserts (Millipore) were placed in a 24-well plate with 600 ​μl of medium containing 10% FBS in the lower chamber. Ten thousand transfected cells in 200 ​μl serum-free medium were added to the upper chamber. After 24 ​h, cells in the upper chamber were removed, and those under the membrane were fixed with methanol, stained with crystal violet for 15 ​min, observed, and counted under a microscope.

### 2.13 Tumor model and In vivo treatments

Animal experiments were conducted in compliance with the ARVO Statement for the Use of Animals in Ophthalmic and Vision Research and approved by the Institutional Animal Care and Use Committee at Zhongshan Ophthalmic Center (O2022051). Female BALB/C nude mice were purchased from Zhuhai BesTest Bio-Tech Co. (Zhuhai, China). Inclusion criteria were predefined as 4-week-old female BALB/C nude mice (16–18g, specific-pathogen-free status) with no visible abnormalities; exclusion criteria included failure to form tumors (volume < 50 ​mm^3^) or severe procedure-related morbidity, though no mice were excluded during the study. A total of 5 ​× ​10^6^ OCM1 cells mixed with Matrigel (Corning, NY, USA) were injected subcutaneously into the right flank of each mouse. Tumor size was measured every 7 days, and volume was calculated using the formula: tumor length (mm) ​× ​tumor width (mm) ​× ​tumor width (mm) ​× ​1/2. At day 7 post-inoculation, the average tumor volume reached approximately 70–80 ​mm^3^, at which point mice were randomly allocated to control (siNC, n ​= ​6) and treatment (siLSM12, n ​= ​6) groups. Tumor-bearing mice received four consecutive intratumoral injections of siNC or siLSM12 (10 nM/20 ​g body weight) using insulin syringe needles, administered once every 7 days starting from day 7 post-inoculation. After 35 days, mice were euthanized by cervical dislocation following isoflurane inhalation.

### 2.14 TUNEL assay

Terminal deoxynucleotidyl transferase-mediated dUTP nick end labeling (TUNEL) assay was performed using the One-step TUNEL In Situ Apoptosis Kit (Green, FITC; Elabscience) following manufacturer's instructions. Paraffin sections were deparaffinized, rehydrated, and washed in phosphate-buffered saline (PBS). Permeabilization was done with 100 ​μL Proteinase K at 37 ​°C for 20 ​min. Sections were equilibrated with 100 ​μL TdT Equilibration Buffer at 37 ​°C for 30 ​min, then incubated with labeling working solution at 37 ​°C in the dark for 60 ​min. After PBS rinses, sections were counterstained with DAPI, washed in PBS, and mounted with anti-fluorescence quenching medium. TUNEL-positive cells relative to DAPI-stained nuclei were counted in random high-power fields (3 independent repetitions per sample).

### 2.15 Statistical analysis

Data are presented as means ​± ​standard deviation (SD). Each cell culture experiment was independently repeated three times with biological replicates to ensure reproducibility. Statistical analyses were performed using GraphPad Prism Software v8.0.2 (GraphPad, La Jolla, CA, USA). Unpaired two-tailed Student's t-test was used for comparisons between two groups, and one-way ANOVA was applied for multiple-group comparisons, followed by Tukey's post-hoc test when significant differences were detected. Exact *P*-values were calculated and reported. In figures, significance levels are denoted as follows: ∗*P* ​< ​0.05, ∗∗*P* ​< ​0.01, ∗∗∗*P* ​< ​0.001, and ∗∗∗∗*P* ​< ​0.0001. A *P*-value threshold of <0.05 was considered statistically significant.

## 3 Results

### 3.1 LSM12 increased expression and its relation to poor prognosis in UM

**Fig. 1:**
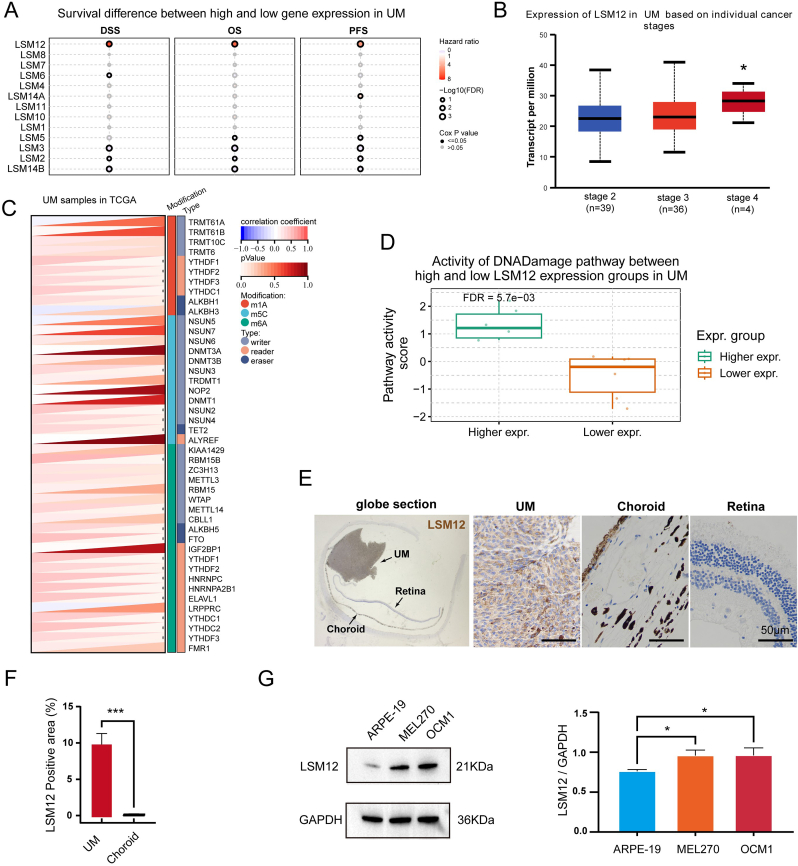
**Increased LSM12 Expression and its Association with Poor Prognosis in Uveal Melanoma. (A)** Survival analysis depicting differences in survival between high and low LSM family gene expression groups in uveal melanoma (UM), conducted using The Cancer Genome Atlas (TCGA) data and the Gene Set Cancer Analysis (GSCA) platform. **(B)** Expression levels of LSM12 in UM across various cancer stages analyzed using individual cancer stage data from UALCAN. **(C)** Analysis of RNA modification genes (m1A, m5C, m6A) and LSM12 expression in UM. **(D)** Activity comparison of the DNA damage pathway between high and low LSM12 expression groups in UM, based on analysis of TCGA data using the Gene Set Cancer Analysis (GSCA) platform. **(E, F)** Immunohistochemistry staining of LSM12 expression in human UM tissues, highlighting the positive area of LSM12 expression in different tissue samples. **(F)** Western blot assays and statistical analysis of LSM12 expression in UM cell lines compared to the control cell line ARPE-19. Data are presented as means ​± ​SD. Statistical significance was determined using Student's t-test and one-way ANOVA, indicated as ∗*P* ​< ​0.05, ∗∗∗*P* ​< ​0.001.

The relationship between LSM family expression and UM prognosis was analyzed using the GSCA database. Among all members, LSM12 showed the strongest association with adverse prognosis, with elevated expression predicting shorter overall survival (OS; HR ​= ​6.33, *P* ​= ​9.01 ​× ​10^−4^), progression-free survival (PFS; HR ​= ​4.80, *P* ​= ​2.89 ​× ​10^−4^), and disease-specific survival (DSS; HR ​= ​7.09, *P* ​= ​1.72 ​× ​10^−3^) (Fig. 1A). UALCAN analysis of TCGA UM cases revealed that stage 4 tumors expressed significantly higher LSM12 levels than stage 3 tumors (*P* ​< ​1 ​× ​10^−12^) (Fig. 1B). Given the important role LSM12 plays in RNA modification, the relationship between LSM12 expression and three types of RNA methylation modifications—m^1^A, m^5^C, and m^6^A—was assessed. The results demonstrated that LSM12 expression was positively correlated with 21 genes related to these RNA methylation modifications (Fig. 1C). Analysis of pathway activity by GSCA revealed that LSM12 might strongly activate the DNA damage response (Fig. 1D). The expression of LSM12 was further validated in UM patient tissue and cell lines. IHC showed that positive LSM12 expression was significantly detected in tumor cells of UM tissue but not in normal choroid and retina (Fig. 1E and F). Western blot assays demonstrated that LSM12 protein expression levels were relatively higher in OCM1 and MEL270 compared to ARPE-19 (Fig. 1G).

### 3.2 LSM12 Knockdown Disrupts UM cell line viability and migration In vitro

**Fig. 2:**
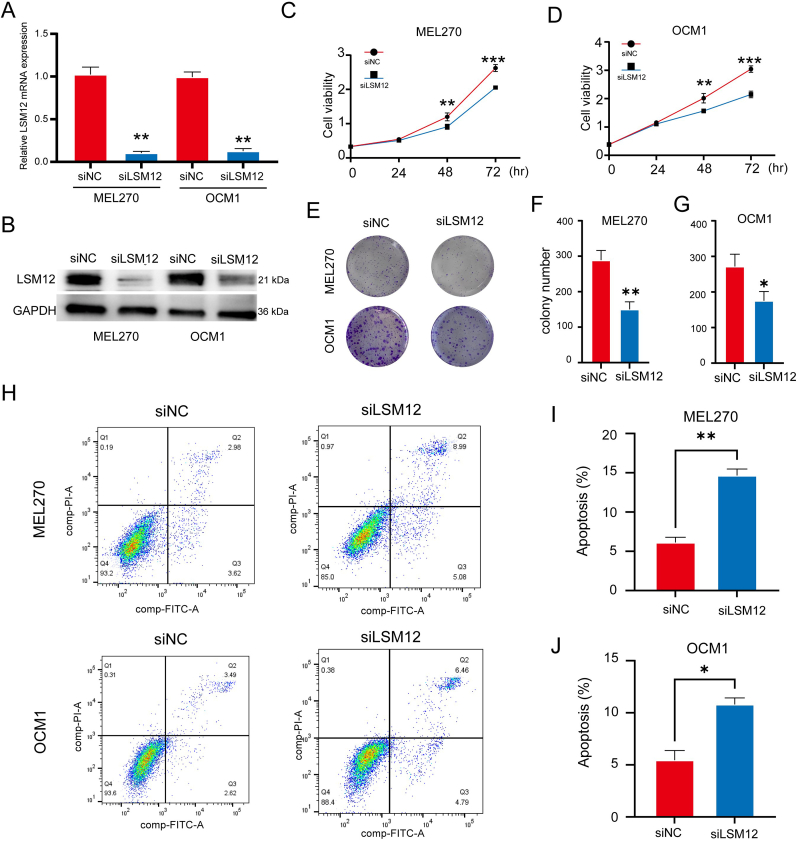
**LSM12 Knockdown Disrupts UM Cell Line Viability In Vitro. (A)** Validation of LSM12 knockdown by qPCR and **(B)** Western blot in UM cell lines Mel270 and OCM1. **(C, D)** CCK-8 analysis showing viability of UM cell lines at 24, 48, and 72 ​h post-LSM12 knockdown with siRNA. **(E**–**G)** Colony formation assay demonstrating the impact of LSM12 knockdown on colony formation ability in Mel270 and OCM1 cell lines, with relative colony numbers presented. **(H**–**J)** Flow cytometry analysis of apoptosis in UM cell lines Mel270 and OCM1 following LSM12 knockdown with siRNA, displaying relative apoptosis cell ratios. Data are presented as means ​± ​SD, with statistical significance assessed by unpaired, two-tailed Student's t-test, ∗*P* ​< ​0.05, ∗∗*P* ​< ​0.01, ∗∗∗*P* ​< ​0.001.

**Fig. 3:**
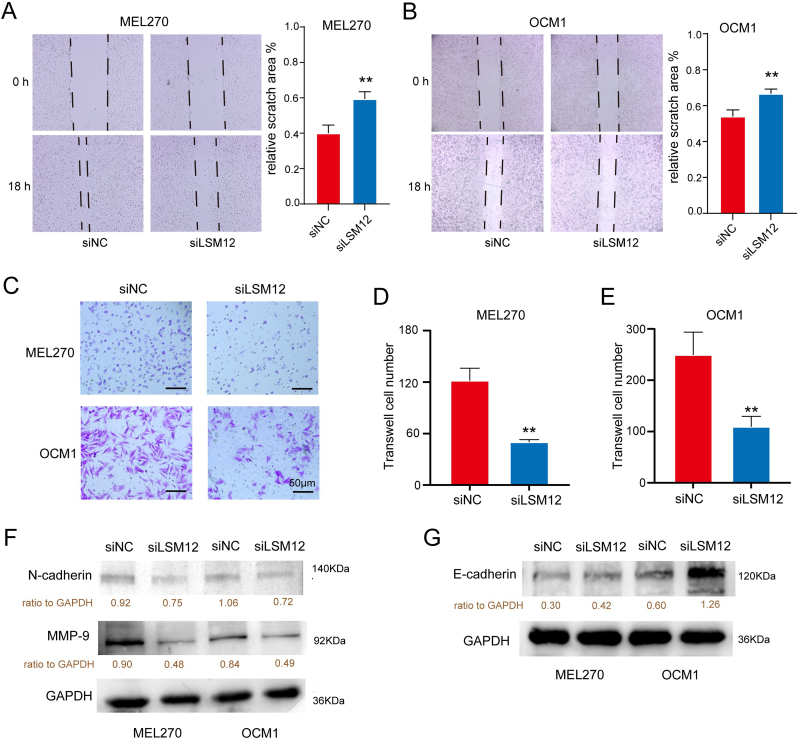
**LSM12 Knockdown Disrupts Migration of UM Cell Lines In Vitro. (A)** Scratch assay of MEL270 ​cells following LSM12 knockdown, with analysis of relative scratch area. **(B)** Scratch assay of OCM1 cells post-LSM12 knockdown, including assessment of relative scratch area. **(C**–**E)** Transwell assay assessing migration of UM cell lines treated with siLSM12, with quantification of migrated cells. **(F)** Western blot analysis showing alterations in N-cadherin and MMP9 protein levels in UM cell lines after LSM12 knockdown. **(G)** Western blot analysis revealing changes in E-cadherin protein expression in UM cell lines following LSM12 knockdown. Data are presented as means ​± ​SD, with statistical significance assessed by unpaired, two-tailed Student's t-test, ∗∗*P* ​< ​0.01.

To explore the role of LSM12 in vitro, siRNA was used to knock down LSM12 expression in OCM1 and MEL270 ​cell lines (Fig. 2A and B). The CCK-8 assay demonstrated a significant reduction in cell viability of OCM1 and MEL270 at 48 and 72 ​h after LSM12 knockdown (Fig. 2C and D). Moreover, the colony formation assay revealed that the ability of OCM1 and MEL270 ​cells to form colonies was markedly diminished following LSM12 knockdown (Fig. 2E–G). Flow cytometry indicated an increased apoptosis ratio in OCM1 and MEL270 ​cells subsequent to LSM12 knockdown (Fig. 2H–J). Cell migration was significantly impaired, as demonstrated by the scratch test (Fig. 3A and B) and the transwell assay (Fig. 3C–E) following LSM12 knockdown. Furthermore, changes in the expression levels of epithelial-mesenchymal transition (EMT)-related markers were examined. Western blot analysis showed a decrease in N-cadherin and MMP9 expression in OCM1 and MEL270 ​cells after LSM12 knockdown, while protein level of E-cadherin was increased (Fig. 3F and G).

### 3.3 LSM12 Knockdown Significantly Inhibits OCM1 xenograft growth

**Fig. 4:**
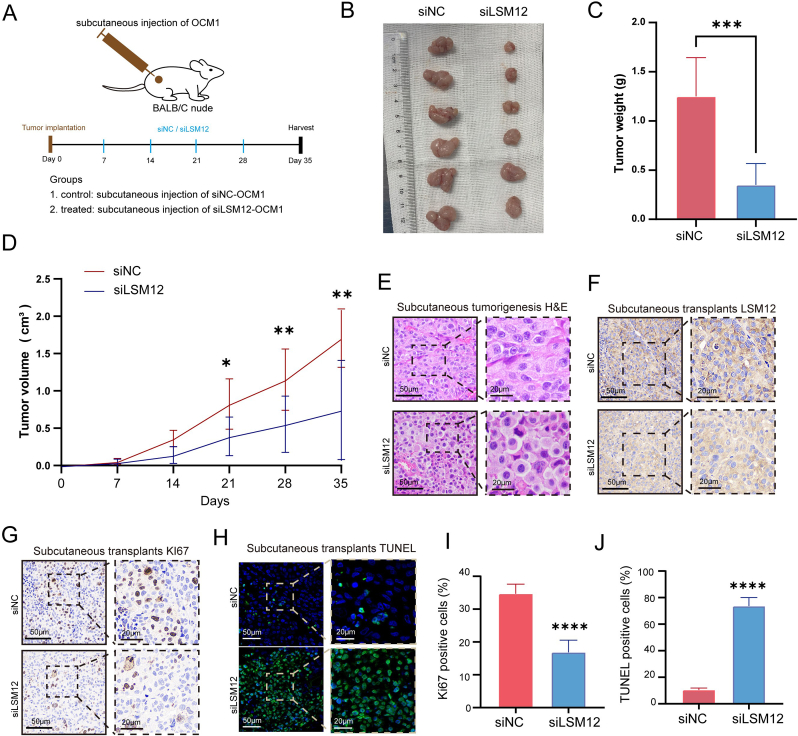
**LSM12 Knockdown Significantly Inhibits OCM1 Xenograft Growth**. **(A)** Experimental workflow diagram. **(B)** External appearance of subcutaneous tumors comparing siNC and siLSM12 groups. **(C)** Tumor weights measured across experimental groups. **(D)** Comparison of tumor volume changes from day 0 to day 35 between siNC and siLSM12 groups. **(E)** Histological analysis using H&E staining showing subcutaneous tumor development in each group. **(F)** Immunohistochemistry (IHC) staining depicting LSM12 expression in subcutaneous xenografts from each group. **(G)** Representative images of Ki67 staining indicating proliferative activity in xenograft tumors. **(H)** Representative images of TUNEL staining showing apoptotic cells in xenograft tumors. **(I, J)** Quantification of Ki67-positive cells and apoptotic cells in xenograft tumors. Data are presented as means ​± ​SD. Statistical significance was determined by unpaired, two-tailed Student's t-test, ∗*P* ​< ​0.05, ∗∗*P* ​< ​0.01, ∗∗∗*P* ​< ​0.001, ∗∗∗∗*P* ​< ​0.0001.

To further elucidate the effects of LSM12 on the tumorigenesis of UM cells in vivo, subcutaneous xenograft experiments were conducted in BALB/c nude mice (Fig. 4A). The tumor growth curves and weight analyses revealed that the tumor volumes and weights in the LSM12 knockdown groups were significantly lower than those in the control groups (Fig. 4B–D). Histological examination confirmed tumor formation in both groups (Fig. 4E), while IHC verified diminished LSM12 expression in the knockdown tumors (Fig. 4F). Functionally, LSM12 depletion decreased proliferative activity, as indicated by reduced Ki-67 staining, and enhanced apoptosis, as shown by TUNEL positivity (Fig. 4G–J). Collectively, these results demonstrate that LSM12 knockdown effectively suppresses OCM1 xenograft growth in vivo.

### 3.4 Association of LSM12 with tumor stemness and the PI3K/Akt/mTOR pathway in UM

**Fig. 5:**
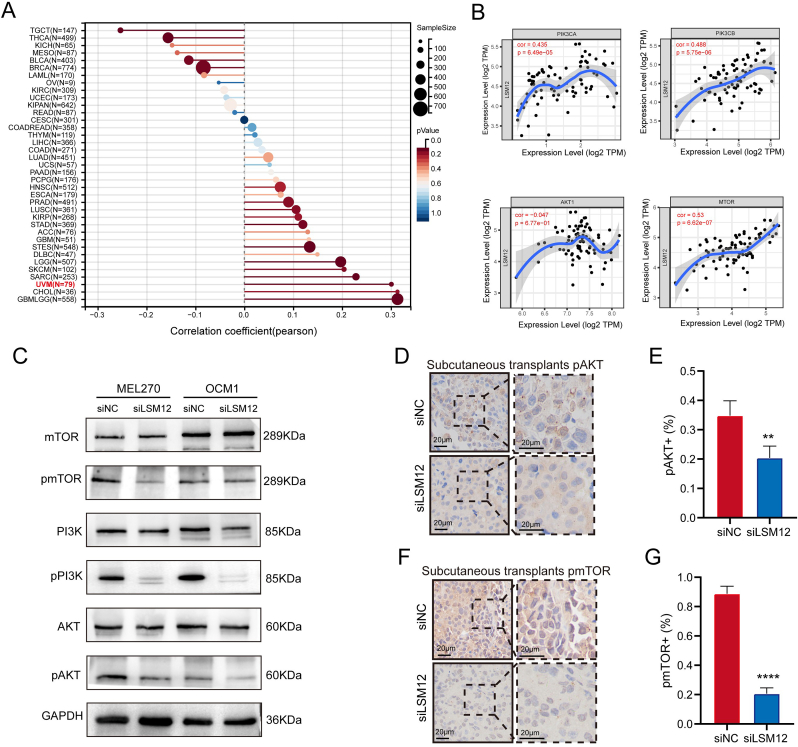
**Association of LSM12 with Tumor Stemness and the PI3K/Akt/mTOR Pathway in UM. (A)** Analysis of the relationship between tumor stemness and LSM12 expression across multiple cancer types using PANCANCER data from TCGA, assessed via the Sangerbox platform. **(B)** Evaluation of potential correlations between LSM12 expression and the PI3K/Akt/mTOR pathway in UM using the TIMER correlation module. **(C)** Western blot assay showing changes in phosphorylation of PI3K/Akt/mTOR pathway proteins after LSM12 knockdown. **(D, E)** Immunohistochemistry (IHC) staining of pAkt in subcutaneous xenografts and statistical analysis of pAkt positivity in each experimental group. **(F, G)** IHC staining of p-mTOR in subcutaneous xenografts and statistical analysis of p-mTOR positivity in each experimental group. Data are presented as means ​± ​SD. Statistical significance was determined by unpaired, two-tailed Student's t-test, ∗∗*P* ​< ​0.01, ∗∗∗∗*P* ​< ​0.0001.

Based on the observed effects of LSM12 knockdown on inhibiting UM cell viability, migration, and in vivo tumorigenesis, an investigation into its potential association with tumor stemness was initiated. Correlation analysis between LSM12 expression and DNA methylation-based stemness scores (DNAss) across various cancer types revealed a significant positive correlation specifically in UM (*R* ​= ​0.30, *P* ​= ​0.0071) (Fig. 5A). The PI3K/Akt/mTOR pathway is a critical signaling pathway involved in maintaining stemness, proliferation, differentiation, and epithelial-mesenchymal transition (EMT) in cancer stem cells (CSCs).^22^, ^23^, ^24^ Data from the "correlation" module of TIMER identified a positive correlation between the mRNA expression levels of LSM12 and PIK3CA, PIK3CB, as well as MTOR, while showing a negative correlation with AKT in UM (Fig. 5B). To further validate the association between LSM12 and the PI3K/Akt/mTOR pathway, Western blot analysis demonstrated reduced expression of phosphorylated AKT (pAKT), PI3K (pPI3K), and MTOR (pMTOR) proteins following LSM12 knockdown in UM cell lines (Fig. 5C). IHC of subcutaneous xenografts corroborated these results, revealing fewer pAKT and pMTOR-positive cells in the siLSM12 group (Fig. 5D–G).

## 4 Discussion

Abnormal epigenetic expression plays a critical role in intraocular tumor development.^6^ The biogenesis and function of mRNA require a series of discrete events such as mRNA processing, transport, translation, and degradation, which are also forms of epigenetic regulation.^25^ LSM proteins impact various aspects of RNA metabolism, among which the RNA-splicing factor LSM12 is involved in nucleocytoplasmic transport, sustaining the Ran (Ras-related nuclear protein) gradient between the cytoplasm and nucleus.^26^ In this study, public database analyses, cellular experiments, and animal models were employed to investigate the role of LSM12 in UM tumorigenesis. The results indicated that increased expression of LSM12 is correlated with poor prognosis in UM. Additionally, LSM12 knockdown disrupted UM cell line viability and migration in vitro and significantly inhibited OCM1 xenograft growth in vivo. LSM12 was associated with tumor stemness and the PI3K/Akt/mTOR pathway in UM, highlighting its potential as a therapeutic target.

The LSM family, comprising 13 members (LSM1 to LSM14B), is strongly linked to tumorigenesis, metastasis, and prognosis in various cancers.^10^^,^^27^^,^^28^ Studies have shown that several LSM members are associated with poor prognosis in different tumors. For instance, LSM2 and LSM4 are significantly correlated with poor overall survival in skin cutaneous melanoma (SKCM) patients,^11^ while LSM1 and LSM4 may serve as prognostic biomarkers in early pancreatic ductal adenocarcinoma (PDAC).^29^ Additionally, LSM5, LSM10, LSM12, and LSM14B reliably predict overall survival in hepatocellular carcinoma (HCC) patients.^9^ However, survival analysis in this study found that only LSM12 is associated with poor prognosis in UM, suggesting a unique molecular role for LSM12 in this cancer. Furthermore, pathway activity analysis indicated that high LSM12 expression in UM correlates with DNA damage, which can induce replication stress and contribute to genome instability, a characteristic feature of both pre-cancerous and cancerous cells.^30^

Several crucial epigenetic alterations have been identified in UM, including but not limited to non-coding RNA aberrations, N6-methyladenosine modifications, local chromosomal remodeling, and histone lactylation.^31^, ^32^, ^33^, ^34^, ^35^ The findings of this study indicate a positive correlation between LSM12 expression and N6-methyladenosine modifications in UM, which is consistent with LSM12's role in RNA processing and metabolism. Despite its potential RNA-binding function, which could broadly impact protein expression, LSM12 has been identified as directly involved in nicotinic acid adenine dinucleotide phosphate (NAADP)-evoked Ca_2_^+^ release.^36^ Significantly, research has indicated the potential involvement of NAADP-mediated Ca_2_^+^ signaling in melanoma.^37^ This highlights the need for further investigation into the specific mechanisms by which LSM12 and NAADP-regulated Ca_2_^+^ signaling contribute to the progression of UM.

Despite significant research and technological progress in recent decades, the treatment of UM remains in need of significant improvement, particularly in addressing metastatic disease. Primary intraocular UM are commonly treated with radiotherapy or enucleation.^38^^,^^39^ However, the prevention and treatment of metastases remain largely inadequate and unsatisfactory. This study investigated the biological function of LSM12, demonstrating that its knockdown significantly inhibited cell growth, colony formation, cell migration, and tumorigenesis in vivo, suggesting that LSM12 could be a potential therapeutic target. However, no specific inhibitors for the LSM protein family are currently available. Advances in RNA therapeutics offer a potential solution. Over the past 50 years, the synthesis of RNA oligonucleotides via phosphoramidite chemistry has led to numerous discoveries and new disease treatments, including antisense oligonucleotides (ASO) and siRNA. Recently, new enzymatic RNA synthesis methods have been developed, producing single-stranded RNA with efficiency and purity comparable to traditional chemical synthesis, without the need for template sequences or the toxic by-products of chemical synthesis.^40^ Notably, the eye presents a uniquely advantageous compartment for localized RNA therapy delivery via topical drops, subconjunctival or intravitreal injection. Compared to systemic administration, localized ocular delivery enhances bioavailability at the target site while minimizing off-target effects. Encouraging clinical responses and favorable safety profiles of RNA therapeutics in several ocular diseases—including glaucoma, dry eye, and retinal disorders—underscore the significant translational potential of this approach.^41^, ^42^, ^43^ However, RNAi technology faces inherent challenges, particularly concerning sequence-dependent off-target effects arising from unintended gene target engagement by longer oligonucleotides. Overall, the localized delivery of siRNA and ASO therapeutics retains potential therapeutic utility in ocular tumors, yet additional research is required to substantiate their efficacy and safety.

The current study has certain limitations. Firstly, the exploration of mechanisms was not comprehensive. While LSM12 was identified as potentially related to UM tumor stem cells, the experiments only investigated changes in the PI3K/Akt/mTOR signaling pathway following LSM12 knockdown. Secondly, the study did not delve further into the specific mechanisms by which LSM12 regulates epigenetic modifications in UM. Additionally, there was limited investigation into the broader network of interactions and pathways that LSM12 might influence. Future studies are needed to comprehensively elucidate the molecular mechanisms underlying LSM12's role in UM tumorigenesis and its potential interactions with other epigenetic regulators.

## 5 Conclusions

In conclusion, this study identifies LSM12 as a critical factor in UM progression and prognosis. 10.13039/100028438Elevated LSM12 expression correlates with poor prognosis and supports UM cell viability, migration, and tumorigenesis. These findings support LSM12 as a potential prognostic biomarker and therapeutic target for UM.

## Study approval

The study was performed in accordance with the Declaration of Helsinki. Human tissue experiments were approved by the Ethics Committee of Zhongshan Ophthalmic Center (Approval No. 2023KYPJ308), and animal experiments were approved by the Institutional Animal Care and Use Committee at Zhongshan Ophthalmic Center (Approval No. O2022051).

## Author contributions

The authors confirm contributions as follows: Conception and design: Yi Ren, Liling Chen. Resources: Yang Gao, Jinmiao Li, Yaoming Liu, Chao Cheng, Ping Zhang, Siming Ai, Yuxiang Mao, Shicai Su, Rong Lu. Data curation: Junjie Tang. Supervision: Rong Lu. Funding acquisition: Yaoming Liu, Rong Lu. Validation: Fengyu Sun, Yi Ren, Yang Gao, Shuxia Chen. Investigation: Junjie Tang, Fengyu Sun, Yi Ren, Liling Chen, Shuxia Chen. Methodology: Fengyu Sun. Project administration: Rong Lu. Writing ​– ​original draft: Junjie Tang. Writing ​– ​review & editing: Junjie Tang, Jinmiao Li, Chao Cheng, Rong Lu. All authors reviewed and approved the final version of the manuscript.

## Funding

This study was supported by the 10.13039/501100001809National Natural Science Foundation of China (Grant Nos. 82471068) and Research Funds of the State Key Laboratory of Ophthalmology, China (Grant Nos. 2025QZLH04).

## Declaration of competing interest

The authors declare that they have no known competing financial interests or personal relationships that could have appeared to influence the work reported in this paper.

## Abbreviations

UM: uveal melanoma
LSM: Smith-like
lncRNAs: long non-coding RNAs
TCGA: The Cancer Genome Atlas
GSCA: Gene Set Cancer Analysis
IHC: immunohistochemistry
qPCR: Quantitative Real-time PCR
siRNA: Small interfering RNA
CCK-8: Cell Counting Kit-8
PFS: progression-free survival
OS: overall survival
EMT: epithelial-mesenchymal transition
DNAss: DNA methylation-based stemness scores
CSCs: cancer stem cells
PDAC: pancreatic ductal adenocarcinoma
HCC: hepatocellular carcinoma
NAADP: nicotinic acid adenine dinucleotide phosphate
ASO: antisense oligonucleotides
