# Supplementary material for: Functional characterization of LSM12 as a driver in uveal melanoma oncogenesis
Source: Adv Ophthalmol Pract Res. 2025 Sep 10;5(4):268–77. doi: 10.1016/j.aopr.2025.09.001 (PMC12509769; doi:10.1016/j.aopr.2025.09.001)
Supplement: Multimedia component 1 [file mmc1.docx]

**Supplementary Material**

**
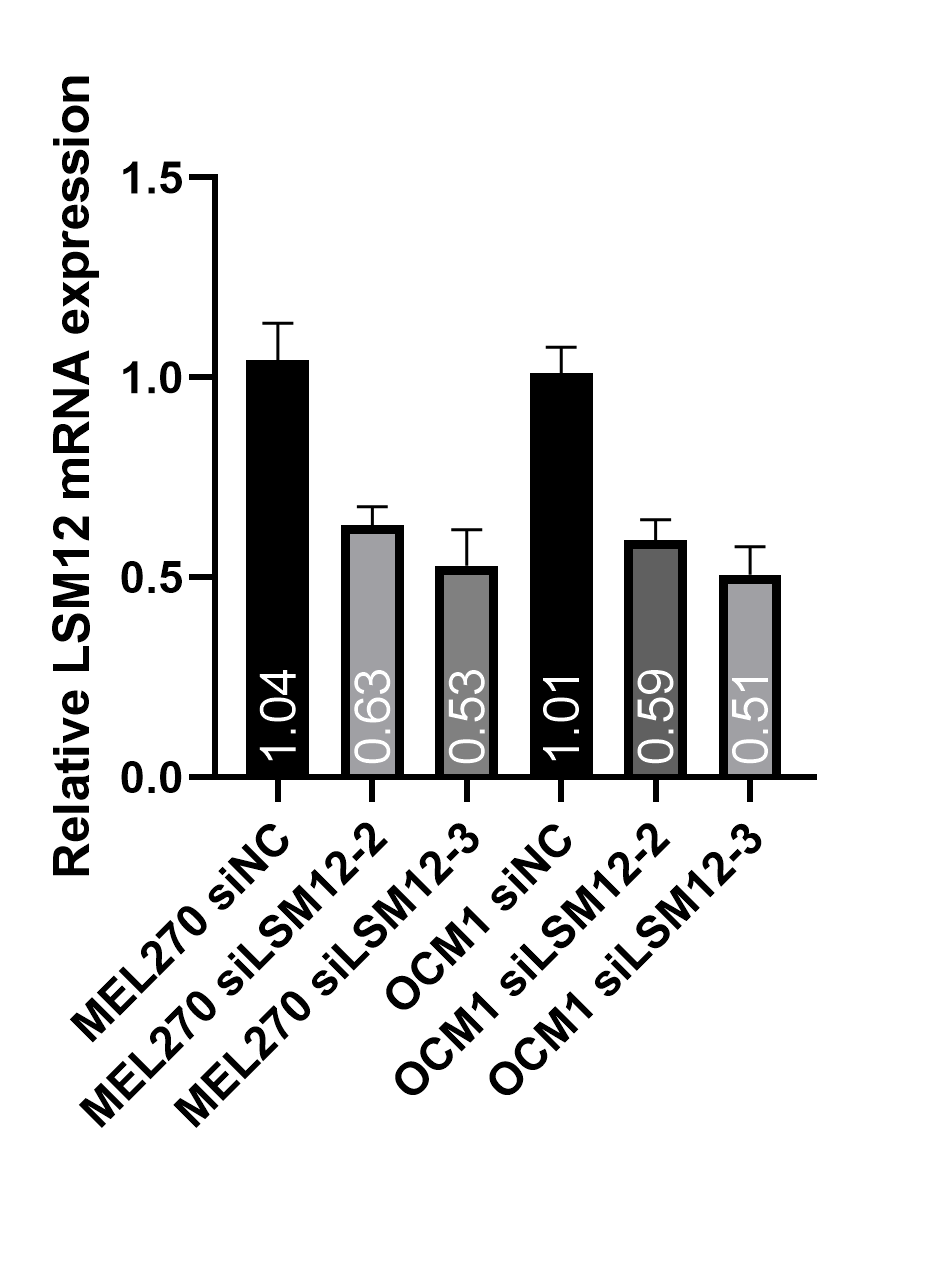
**

| siLSM12-2 | Sense: 5’-*GCAAGAAGAUGGUUAUCAUU*-3’  Antisense: 5’-*UGAUACC AUCUUCUUGCUUU*-3’ |
| --- | --- |
| siLSM12-3 | Sense: 5’-*GGAGAUUGUUGAAUUGGAUUU*-3’  Antisense: 5’-*UCCAAUUCAACAUCUCCUU*-3’ |

**Figure S1. Alternative siRNA sequences targeting LSM12**

**
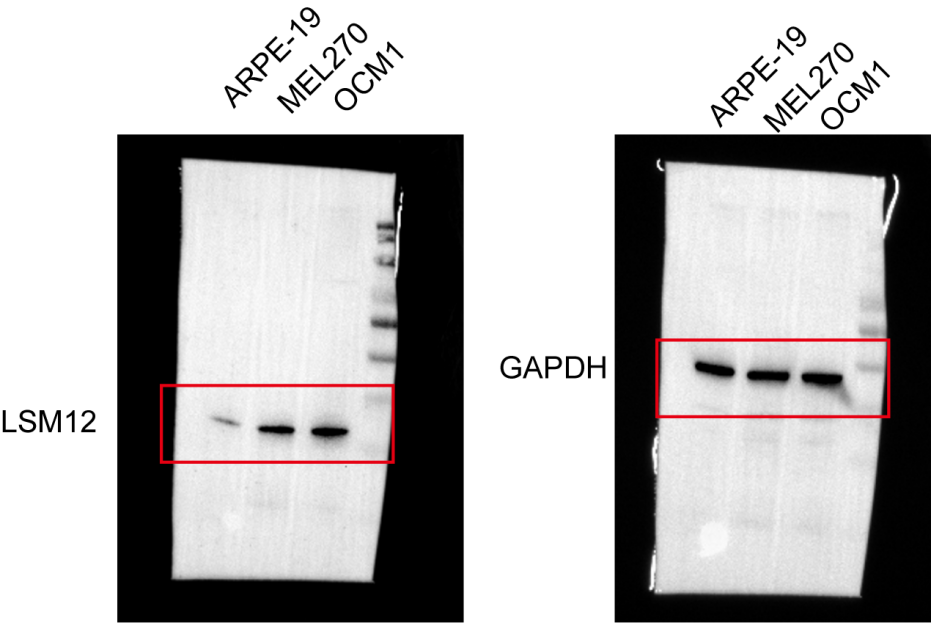
**

**Figure S2. Uncropped original western blots related to Figure 1 G.**

**
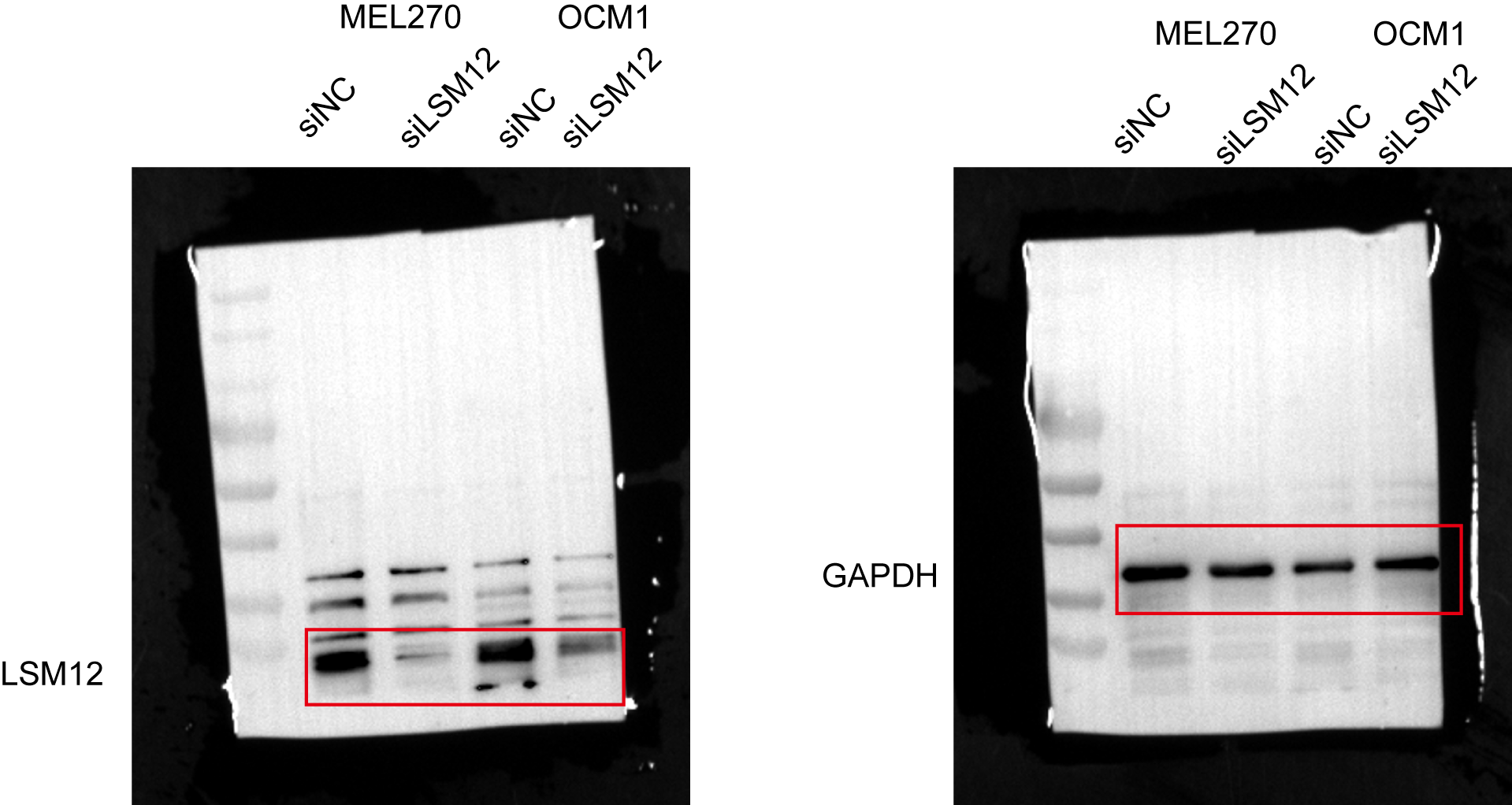
**

**Figure S3. Uncropped original western blots related to Figure 2 B.**

**
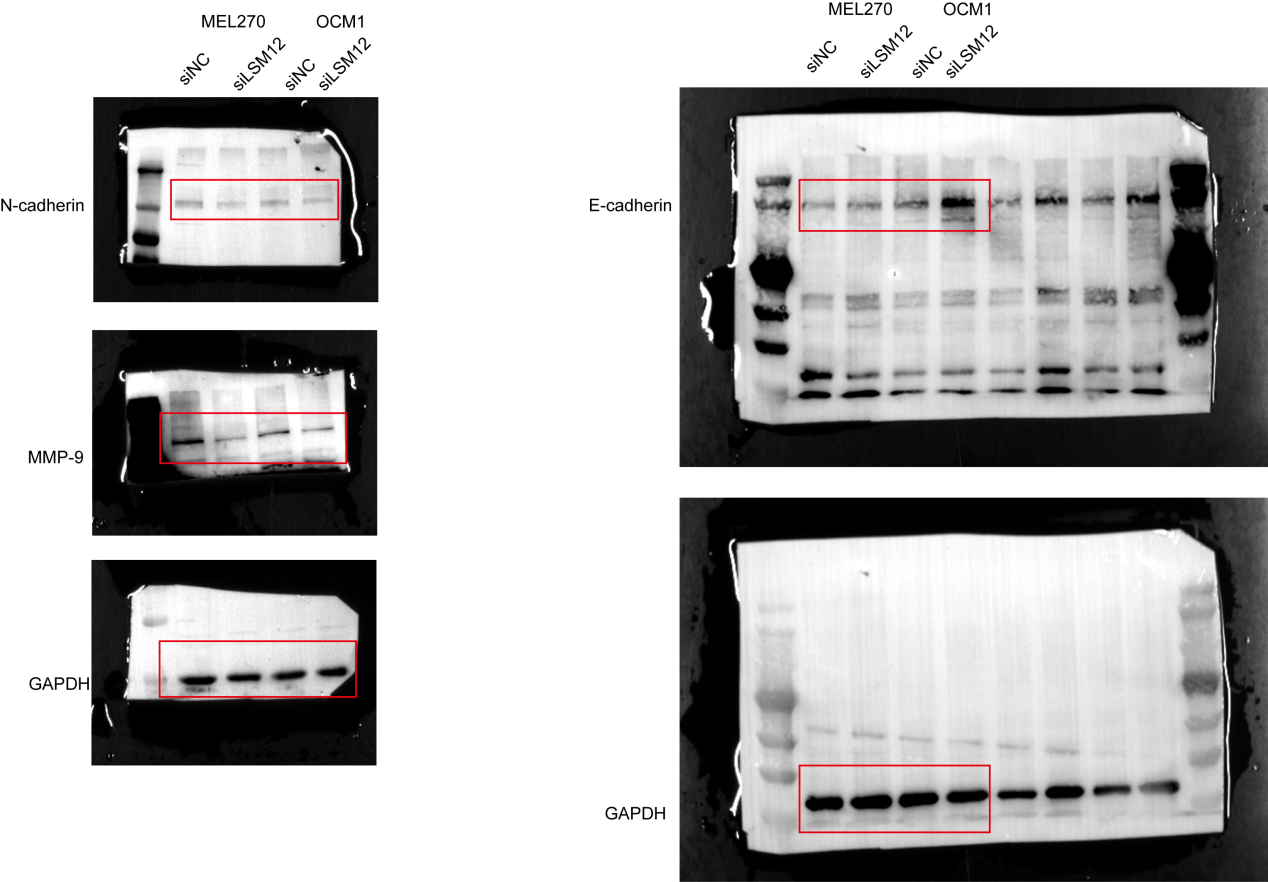
**

**Figure S4. Uncropped original western blots related to Figure 3 F, G.**

**
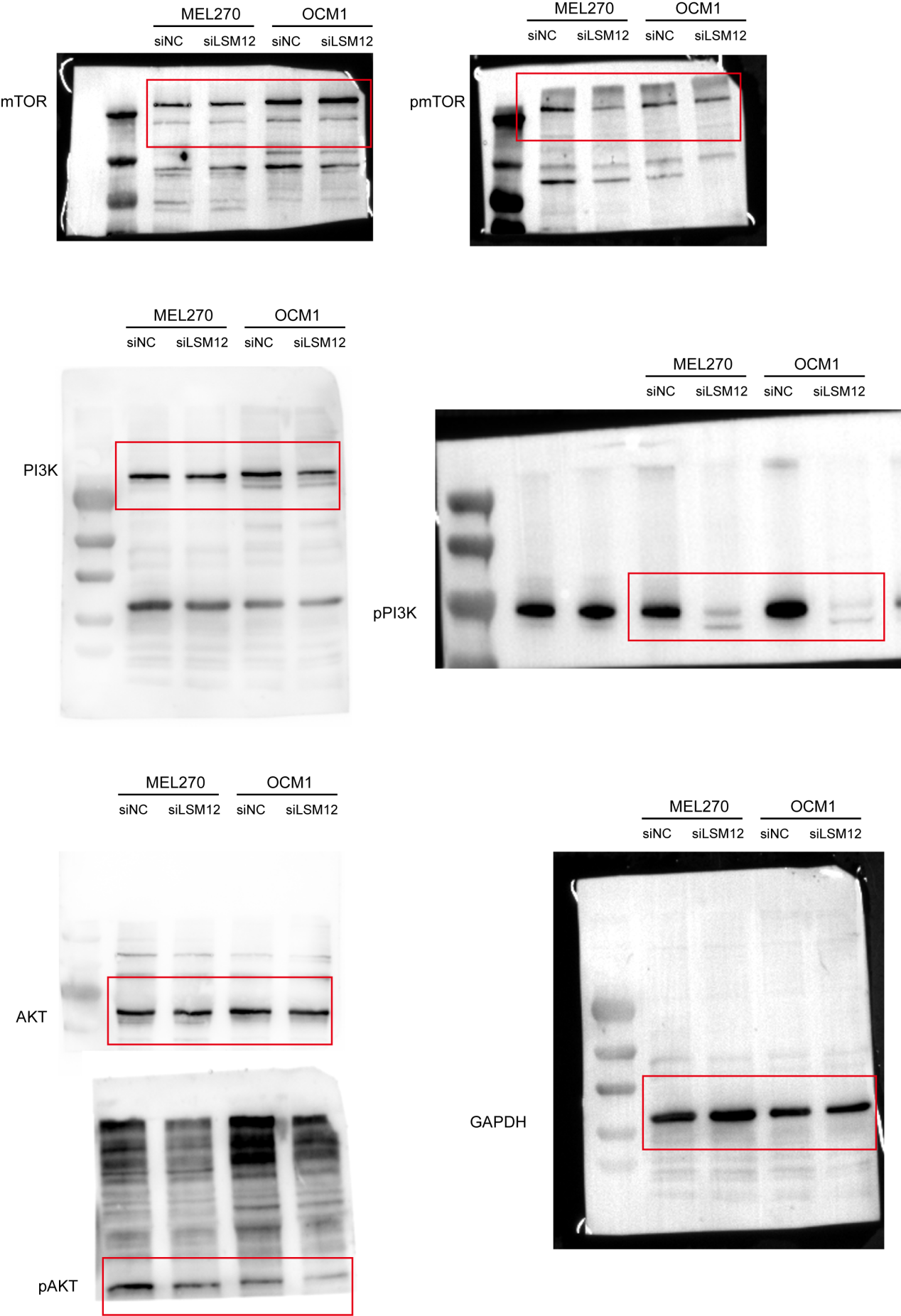
**

**Figure S5. Uncropped original western blots related to Figure 5 C.**
